# Alterations of sorbin and SH3 domain containing 3 (SORBS3) in human skeletal muscle following Roux-en-Y gastric bypass surgery

**DOI:** 10.1186/s13148-017-0396-5

**Published:** 2017-09-02

**Authors:** Samantha E. Day, Luis A. Garcia, Richard L. Coletta, Latoya E. Campbell, Tonya R. Benjamin, Elena A. De Filippis, James A. Madura, Lawrence J. Mandarino, Lori R. Roust, Dawn K. Coletta

**Affiliations:** 1 School of Life Sciences, Arizona State University, Tempe, AZ USA; 2 Department of Medicine, The University of Arizona College of Medicine, PO Box 245035, 1501 N. Campbell Ave, Tucson, AZ 85724-5035 USA; 3 Endocrinology Department, Mayo Clinic in Arizona, Scottsdale, AZ USA; 4 Department of Basic Medical Sciences, The University of Arizona College of Medicine – Phoenix, Phoenix, AZ USA

**Keywords:** DNA methylation, Next-generation sequencing, Skeletal muscle, Obesity, Surgery

## Abstract

**Background:**

Obesity is a disease that is caused by genetic and environmental factors. However, epigenetic mechanisms of obesity are less well known. DNA methylation provides a mechanism whereby environmental factors can influence gene transcription. The aim of our study was to investigate skeletal muscle DNA methylation of sorbin and SH3 domain containing 3 (*SORBS3*) with weight loss induced by Roux-en-Y gastric bypass (RYGB).

**Results:**

Previously, we had shown increased methylation (5.0 to 24.4%) and decreased gene expression (fold change − 1.9) of *SORBS3* with obesity (BMI > 30 kg/m^2^) compared to lean controls. In the present study, basal muscle biopsies were obtained from seven morbidly obese (BMI > 40 kg/m^2^) female subjects pre- and 3 months post-RYGB surgery, in combination with euglycemic-hyperinsulinemic clamps to assess insulin sensitivity. We identified 30 significantly altered promoter and untranslated region methylation sites in *SORBS3* using reduced representation bisulfite sequencing (RRBS). Twenty-nine of these sites were decreased (− 5.6 to − 24.2%) post-RYGB compared to pre-RYGB. We confirmed the methylation in 2 (Chr.8:22,423,690 and Chr.8:22,423,702) of the 29 decreased *SORBS3* sites using pyrosequencing. This decreased methylation was associated with an increase in *SORBS3* gene expression (fold change + 1.7) post-surgery. In addition, we demonstrated that *SORBS3* promoter methylation in vitro significantly alters reporter gene expression (*P* < 0.0001). Two of the *SORBS3* methylation sites (Chr.8:22,423,111 and Chr.8:22,423,205) were strongly correlated with fasting plasma glucose levels (*r* = 0.9, *P* = 0.00009 and *r* = 0.8, *P* = 0.0010). Changes in *SORBS3* gene expression post-surgery were correlated with obesity measures and fasting insulin levels (*r* = 0.5 to 0.8; *P* < 0.05).

**Conclusions:**

These results demonstrate that *SORBS3* methylation and gene expression are altered in obesity and restored to normal levels through weight loss induced by RYGB surgery.

## Background

One third of the US adult population is obese (body mass index [BMI] > 30 kg/m^2^), and the number of individuals entering into morbid obesity (BMI > 40 kg/m^2^) is on the rise [1, 2]. Roux-en-Y gastric bypass (RYGB) is one of the most common surgeries performed to treat obesity and combines restrictive and malabsorptive techniques [3]. Besides weight loss, other benefits of surgical intervention have included improved blood glucose levels and insulin sensitivity and secretion [3–5]. However, these studies do not completely explain the molecular basis of the metabolic improvements observed with weight loss induced by surgery.

One of the most studied epigenetic marks is DNA methylation, which is the addition of a methyl group to the fifth carbon of a cytosine, typically preceding a guanine, termed CpG dinucleotide [6]. The addition or removal of these marks has regulatory influence on gene expression [7]. Our previously published study identified a novel gene, sorbin and SH3 domain containing 3 (*SORBS3*), that was differentially methylated with obesity [8]. Specifically, we had shown an increase in skeletal muscle promoter methylation and a decrease in mRNA expression of *SORBS3* with obesity [8]. The *SORBS3* gene codes for the adapter protein vinexin and has been shown to play a role in growth-factor-induced signal transduction and cytoskeleton structure [9]. We have previously proposed a model where chronic inflammation from obesity may lead to insulin resistance by inducing changes to the extracellular matrix that are reminiscent of fibrosis and alter mechanosignal transduction mediated by cytoskeletal elements [10]. We believe that changes in *SORBS3* expression may be connected to our proposed model since it is a cytoskeletal gene that was reduced with obesity [8]. However, it is unclear whether the reduction in *SORBS3* observed in obesity could be rescued through surgical weight-loss interventions such as RYGB.

Here, we set out to determine if the changes in *SORBS3* DNA methylation identified in obesity and its underlying insulin resistance can be altered in response to weight loss, using our previously described RYGB surgery cohort [11]. We hypothesized that 3 months post-surgery, *SORBS3* methylation would be decreased and this would result in an increased gene expression, so it would be normalized to levels observed in lean controls.

## Methods

### Study design

Seven (one of which was diabetic, treated with metformin) morbidly obese (BMI > 40 kg/m^2^) females (ages 33–59 years) participated in this study pre- and 3 months post-RYGB surgery. The study was approved by the institutional review boards at the Mayo Clinic in Arizona and Arizona State University. The design of this study and surgical procedure have been previously described [11].

### Muscle biopsy processing

Genomic DNA and total RNA were isolated from muscle biopsies as previously described [8].

### Reduced representation bisulfite sequencing (RRBS)

RRBS sample preparation was performed on the pre- and 3-month post-surgery DNA by the Mayo Clinic Genotyping Shared Resource facility. The RRBS sample preparation has been described in detail elsewhere [8]. Sequence data was processed using the streamlined analysis and annotation pipeline for reduced representation bisulfite sequencing, SAAP-RRBS [8, 12].

### Differentially methylated cytosine (DMC) analysis pre- and post-surgery

Differences in methylation sites were assessed in participants pre- and post-surgery. The aligned (Hg19) sequencing data was imported into the free open source R package, methylSig [13]. A minimum of five reads and the recovery of the site in all seven participants from pre- and post-surgery were required for the inclusion of a cytosine in subsequent analyses. The mean methylation differences (%) were determined and annotations were applied, as previously described [8]. DMCs within the promoter and untranslated regions for *SORBS3* were extracted from the data set.

### SORBS3 pyrosequencing

DNA methylation sites were confirmed using pyrosequencing, as previously described [8]. To assess *SORBS3* DMCs at positions Chr.8:22,423,519 and Chr.8:22,423,529 on the sense strand, bisulfite-converted DNA was amplified by PCR using the following primers: forward 5′-AGTAGGGGGAGGAAGGAA-3′ and biotinylated reverse 5′-ACCCCCATCCTCTACTAAAAATTAAC-3′. For the DMCs at positions Chr.8:22,423,690 and Chr.8:22,423,702 on the antisense strand, bisulfite-converted DNA was amplified by PCR using the following primers: forward 5′-GGGTTTTGGGTTTTTTATAGGATG-3′ and biotinylated reverse 5′- CCACCCAAAACAACTAACTCCTAAC-3′. Pyrosequencing was performed using the PyroMark Q96 MD system and the Gold Q96 kit with sequencing primers for the sense 5′-GGGGGAGGAAGGAAT-3′ and antisense 5′-TGGGTTTTTTATAGGATGT-3′ strands according to the manufacturer’s instructions (Qiagen, Valencia, CA). Sequence analysis was performed using the PyroMark CpG SW 1.0 software (Qiagen, Valencia, CA).

### SORBS3 quantitative real-time PCR (qRT-PCR)

Gene expression for *SORBS3* pre- and post-surgery was detected using qRT-PCR on the ABI PRISM 7900HT sequence detection system (Life Technologies, Carlsbad, CA). The qRT-PCR analyses of the samples were performed using the TaqMan primer and probes as previously described [8].

### Luciferase assay

An 811 bp fragment of the human *SORBS3* promoter (Chr8:22,422,247–22,423,057) was cloned into a CpG-free luciferase reporter vector (pCpGL-basic). The *SORBS3* construct was either mock methylated or methylated using 1600 μM S-adenosylmethionine (SAM) and two different DNA methyltransferases, SssI and HhaI (New England Biolabs, Frankfurt, Germany). Mouse muscle cell lines C2C12 were cultured in DMEM, supplemented with 10% serum and 1% of an antibiotic/antimycotic mixture. Cells were co-transfected with 100 ng of pCpGL-basic with the *SORBS3* promoter insert or without (control) and 2 ng of pRL renilla luciferase control reporter vector using the Lipofectamine 3000 transfection reagent (Invitrogen, Carlsbad, CA). Firefly luciferase activity was measured and normalized against the renilla luciferase activity using the Dual Luciferase Reporter Assay System (Promega, Madison, WI). The results presented are a mean of four independent experiments, containing the mean of five replicates in each experiment.

### SORBS3 comparative DMC analysis

The RRBS data from our previous lean versus obese participant study [8] was used for comparative analysis. The data comprised of 11 lean (ages 21–43 years; 7 females/4 males; BMI 23.4 ± 2.4 kg/m^2^) and 9 obese (ages 32–52 years; 4 females/5 males; BMI 32.9 ± 2.3 kg/m^2^) participants.

### Predictive transcription factor binding analysis

PROMO version 3.0.2 was used to perform transcription factor binding site analysis [14]. Sequences were analyzed with a 5% maximum matrix dissimilarity rate using TRANSFAC version 8.3 database. Analysis of the 30 *SORBS3* DMCs was assessed as 10 separate sequences: Chr.8:22,411,723–22,411,734; Chr.8:22,422,932–22,422,973; Chr.8:22,423,009–22,423,025; Chr.8:22,423,086–22,423,116; Chr.8:22,423,181–22,423,215; Chr.8:22,423,219–22,423,256; Chr.8:22,423,514–22,423,573; Chr.8:22,423,684–22,423,695; Chr.8:22,423,697–22,423,741; and Chr.8:22,423,769–22,423,857.

### Statistical analysis

Pre- and post-surgery comparisons were based on a paired Student *t* test. All phenotypic data was normally distributed and presented as a mean ± standard deviation (SD). Pearson correlation analysis was performed to determine the relationship between DNA methylation from RRBS or gene expression and the phenotypic data. A Bonferroni correction was applied to the Pearson correlation analysis performed on the RRBS 30 DMCs with the phenotype data. Therefore, for the correlation analysis of the RRBS DMCs with the phenotypic data, we considered a *P* ≤ 0.0167 to be significant. For all other correlations, we used the *P* ≤ 0.05 cutoff. See above for the statistical analysis of the methylation and qRT-PCR data.

## Results

### Participants

The metabolic data for these subjects have been described in a previous publication [11]. Briefly, 3 months post-surgery, significant improvements were observed (Table 1) in BMI, body fat percentage, cholesterol, low-density lipoprotein (LDL), fasting plasma glucose (FPG), fasting serum insulin (FSI), and homeostatic model assessment for insulin resistance (HOMA-IR). However, there were no significant improvements observed in blood pressure, triglycerides, high-density lipoprotein (HDL), hemoglobin A1c (HbA1c), endogenous glucose production (EGP), and insulin-stimulated glucose disposal (M-value).

**Table 1:** Phenotype data pre- and 3 months post-Roux-en-Y gastric bypass surgery

	Pre-surgery obese	Post-surgery obese	*P* value
			Pre vs. post
Sex	7 female	7 female	–
Age (years)	45.1 ± 9.4	45.3 ± 9.3	NS
Body mass index (kg/m^2^)	42.1 ± 5.9	35.3 ± 4.9	< 0.001
Body fat (%)	46.4 ± 3.2	40.6 ± 3.4	< 0.01
Systolic blood pressure (mmHg)	125.1 ± 10.3	119.1 ± 12.2	NS
Diastolic blood pressure (mmHg)	71.7 ± 5.4	75.1 ± 4.4	NS
Triglycerides (mg/dL)	121.9 ± 46.2	107.7 ± 29.5	NS
Cholesterol (mg/dL)	181.4 ± 34.9	151.5 ± 29.6	< 0.01
High-density lipoprotein (mg/dL)	45.0 ± 7.1	45.0 ± 6.7	NS
Low-density lipoprotein (mg/dL)	112.1 ± 31.6	84.8 ± 27.8	< 0.01
Hemoglobin A1c (%)	6.0 ± 0.4	5.7 ± 0.3	NS
Fasting plasma glucose (mg/dL)	104.2 ± 20.7	86.7 ± 8.2	< 0.05
Fasting plasma insulin (μU/mL)	18.2 ± 10.1	7.5 ± 4.2	< 0.01
EGP (mg/kg/min)	1.5 ± 0.1	1.5 ± 0.1	NS
*M*-value (mg/kg/min)	2.4 ± 0.9	2.9 ± 1.0	NS
*M*-value (mg/kgFFM/min)	4.4 ± 1.7	4.9 ± 1.6	NS
HOMA-IR	4.4 ± 2.2	1.6 ± 0.9	< 0.05

Data presented as mean ± SD

*HOMA-IR* homeostatic model assessment for insulin resistance, *EGP* endogenous glucose production

### SORBS3 differentially methylated cytosines (DMCs)

Methylation sites within the promoter (0 to − 1000 base pairs from transcription start site) and untranslated regions (5′ and 3′UTR) were used to detect sites that may lead to a change in *SORBS3* mRNA expression. From the RRBS, 352 CpG sites associated with SORBS3 were captured. Of these sites, 8.5% were differentially methylated (30 DMCs). From the 30 DMCs, there were 20 DMCs in the sense strand and 10 DMCs in the antisense strand identified (Table 2). As shown in Table 2, 29 of the 30 sites were decreased post-RYGB. The loss of DNA methylation post-surgery was detected specifically for *SORBS3* and was not the by-product of global losses in methylation (mean ± standard deviation, global pre 33.9 ± 39.9% versus global post 33.7 ± 39.7%).

**Table 2:** Differentially methylated cytosines (DMC; *P* < 0.05) post-surgery that were associated with *SORBS3*

	DNA methylation (%)			Total no. of reads						
Chr.8 position	Pre-surgery	Post-surgery	*P* value	Pre-surgery	Post-surgery	Strand	Gene region	CpG island region	Correlated GE	TFBM overlap
22,411,728	16.3 ± 5.8	7.0 ± 6.0	0.03	135.0	133.0	+	5′UTR	South shelf		
22,411,729	6.6 ± 7.9	0.0 ± 0.0	0.005	89.0	106.0	−	5′UTR	South shelf		
22,422,937	19.4 ± 25.1	5.8 ± 7.8	0.04	125.0	194.0	−	Promoter	CpG island		AP-2alphaA
22,422,940	27.8 ± 24.8	10.2 ± 12.2	0.02	125.0	194.0	−	Promoter	CpG island		
22,422,953	35.2 ± 22.2	11.2 ± 12.4	0.003	129.0	200.0	−	Promoter	CpG island		Sp1
22,422,968	16.5 ± 10.6	5.3 ± 8.5	0.007	129.0	200.0	−	Promoter	CpG island		
22,423,014	21.4 ± 14.6	7.7 ± 5.6	0.003	118.0	120.0	+	Promoter	CpG island		GCF
22,423,020	18.0 ± 7.8	7.5 ± 7.9	0.04	118.0	120.0	+	Promoter	CpG island		GCF
22,423,091	22.4 ± 11.2	7.9 ± 6.5	0.006	119.0	120.0	+	Promoter	CpG island		
22,423,100	17.2 ± 10.8	7.1 ± 5.7	0.03	119.0	120.0	+	Promoter	CpG island		CREB
22,423,111	10.7 ± 12.2	1.3 ± 2.1	0.02	119.0	120.0	+	Promoter	CpG island		
22,423,186	8.8 ± 12.5	22.4 ± 11.3	0.04	84.0	103.0	+	5′UTR	CpG island		
22,423,198	13.8 ± 8.7	5.0 ± 3.9	0.03	130.0	158.0	+	5′UTR	CpG island		
22,423,202	18.8 ± 13.2	8.8 ± 2.4	0.01	130.0	158.0	+	5′UTR	CpG island		
22,423,204	7.9 ± 6.0	0.0 ± 0.0	0.0001	130.0	158.0	+	5′UTR	CpG island		GCF
22,423,205	8.4 ± 5.4	1.9 ± 2.1	0.001	258.0	290.0	−	5′UTR	CpG island		GCF
22,423,206	14.7 ± 7.7	6.9 ± 4.8	0.03	130.0	158.0	+	5′UTR	CpG island		GCF
22,423,210	23.7 ± 11.2	14.1 ± 3.1	0.03	130.0	158.0	+	5′UTR	CpG island		GCF
22,423,224	13.1 ± 8.5	3.3 ± 3.9	0.001	187.0	248.0	+	5′UTR	CpG island		
22,423,235	17.4 ± 11.4	10.1 ± 3.0	0.04	228.0	289.0	+	5′UTR	CpG island		Sp1/Pax5/p53
22,423,251	18.2 ± 12.1	8.4 ± 5.4	0.04	130.0	158.0	+	5′UTR	CpG island		
22,423,519	28.0 ± 8.5	14.3 ± 5.8	0.002	193.0	216.0	+	5′UTR	CpG island	X	
22,423,529	29.8 ± 6.5	17.9 ± 8.1	0.005	184.0	207.0	+	5′UTR	CpG island		
22,423,568	26.5 ± 9.0	16.0 ± 8.7	0.02	359.0	415.0	+	5′UTR	CpG island		
22,423,689	46.8 ± 9.8	31.1 ± 13.6	0.01	252.0	310.0	+	5′UTR	CpG island	X	GCF
22,423,690	50.6 ± 6.8	38.0 ± 16.5	0.04	276.0	342.0	−	5′UTR	CpG island		GCF
22,423,702	38.4 ± 10.7	20.9 ± 14.5	0.007	278.0	345.0	−	5′UTR	CpG island	X	
22,423,736	18.5 ± 5.6	11.2 ± 4.4	0.007	363.0	441.0	+	5′UTR	South shore		RXR-alpha
22,423,774	53.0 ± 14.2	32.1 ± 22.5	0.03	101.0	79.0	−	5′UTR	South shore		
22,423,852	31.2 ± 11.9	15.0 ± 12.7	0.03	92.0	73.0	−	5′UTR	South shore		

DNA methylation data presented as mean ± SD

*GE* correlated gene expression, *TFBM* transcription factor binding motif

### SORBS3 validation

**Fig. 1:**
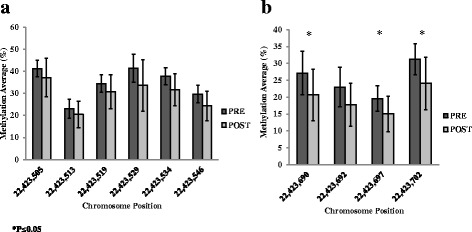
Differentially methylated cytosines (DMCs) associated with *SORBS3* detected using pyrosequencing on the sense strand (**a**) and antisense strand (**b**) pre- and post-surgery. Data presented as mean ± SD

Pyrosequencing was used for confirmation of the 30 *SORBS3* DMCs where primers could be designed. The *SORBS3* region that we were targeting for pyrosequencing was difficult to validate. The sequence characteristics of that region resulted in amplicons that would be too long or would have too many CpG sites and as such resulted in the failure of the assay design. Regardless, we were able to design primers that captured the DMCs at positions Chr.8:22,423,519 and Chr.8:22,423,529 on the sense strand, as well as four additional CpG sites. All six sites were trending towards a decrease in methylation post-surgery; however, none were significant (Fig. 1a). The sequence that encompassed the DMCs at positions Chr.8:22,423,690 and Chr.8:22,423,702 on the antisense strand included two additional CpG sites. All four sites were decreased in methylation post-surgery, and changes in three of these sites were statistically significant (*P* < 0.05; Fig. 1b).

### SORBS3 gene expression

The qRT-PCR results demonstrated an increase in gene expression of *SORBS3* post-surgery compared to pre-surgery (+ 1.7 versus 1.0 fold change, respectively; *P* = 0.05).

### SORBS3 correlation analysis

Pearson correlation analysis was performed to determine the relationship between the phenotypic data and *SORBS3* changes observed with surgery. Of the DMCs detected using RRBS, we demonstrated two Bonferroni-corrected significant associations with fasting plasma glucose (FPG) at DMCs Chr.8:22,423,111 (*r* = 0.9, *P* = 0.00009) and Chr.8:22,423,205 (*r* = 0.8, *P* = 0.0010). Association of *SORBS3* gene expression with phenotypic data was performed using the Ct values from qRT-PCR. Pearson’s correlation analysis identified a significant relationship between the gene expression data and BMI (*r* = 0.8, *P* = 0.00040), percent body fat (*r* = 0.6, *P* = 0.02), and FSI (*r* = 0.5, *P* = 0.04). Furthermore, an association between *SORBS3* gene expression and methylation was identified at DMCs Chr.8:22,423,519 (*r* = 0.7, *P* = 0.004), Chr.8:22,423,689 (*r* = 0.5, *P* = 0.05), and Chr.8:22,423,702 (*r* = 0.6, *P* = 0.03).

### SORBS3 promoter methylation in vitro alters reporter gene expression

**Fig. 2:**
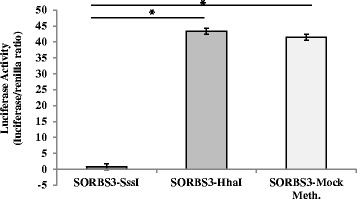
In vitro DNA methylation of the *SORBS3* human promoter is associated with decreased gene expression. Data presented as mean ± SD. The mean represents four independent experiments with five replicates per experiment. *P < 0.0001

The *SORBS3* construct was created to test the effect of DNA methylation on transcriptional activity. Level of suppressed transcriptional activity, as measured by luciferase activity, was determined in comparison to the mock methylated control (Fig. 2). As shown in Fig. 2, when the *SORBS3* construct was methylated in vitro using the HhaI enzyme (GCGC, *n* = 8 sites), transcriptional activity was not suppressed but was significantly suppressed with the SssI enzyme methylation (CG, *n* = 59 sites).

### Predicted transcription factor binding analysis

To identify potential transcription factor binding that may be inhibited by *SORBS3* methylation, we analyzed sequences containing DMCs using PROMO [14]. Transcription factor binding motifs were identified to overlap 13 of the 30 DMCs for *SORBS3*: Chr.8:22,422,937: AP-2alphaA; Chr.8:22,422,953: Sp1; Chr.8:22,423,014 and 22,423,020: GCF; Chr.8:22,423,100: CREB; Chr.8:22,423,204, 22,423,205, 22,423,206, and 22,423,210: GCF; Chr.8:22,423,235: Sp1, Pax-5, and p53; Chr.8:22,423,689 and 22,423,690: GCF; and Chr.8:22,423,736: RXR-alpha.

### SORBS3 alterations with obesity and RYGB surgery

**Fig. 3:**
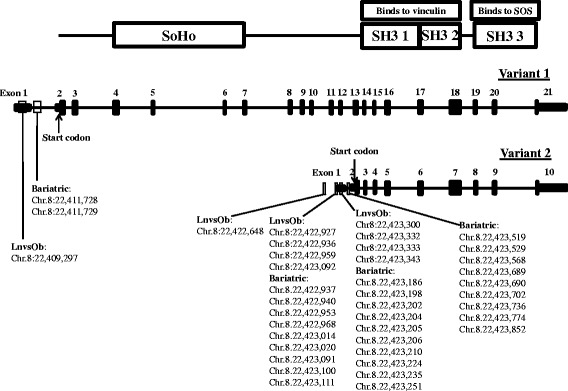
Differentially methylated cytosine (DMC) distribution among the promoter and 5′ untranslated regions of sorbin and SH3 domain containing 3 (*SORBS3*) variants 1 and 2. The DMCs are derived from a previous study in obesity (Ln = lean vs Ob = obese) and the present RYGB cohort (bariatric)

**Fig. 4:**
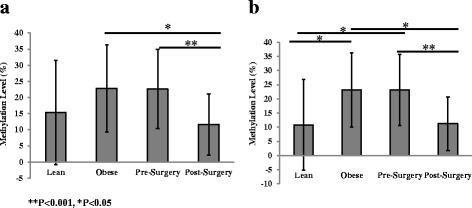
Average methylation levels of *SORBS3* DMCs from lean and obese participants in a previous study and the present study pre- and post-surgery levels. The average methylation was assessed with all DMCs, regardless of methylation direction (**a**) and of only the DMCs that were consistent in the direction of methylation (**b**). Data presented as mean ± SD

Increased DNA methylation in the promoter and 5′UTR of *SORBS3* with obesity were originally identified in our previous study [8]. In the RYGB cohort, methylation levels of *SORBS3* were found to decrease post-surgery. Upon comparing the 10 DMCs (9 increased and 1 decreased) from our previous study, and the 30 DMCs (29 decreased and 1 increased) identified with RYGB surgery, we found sites to cluster in the same region, but no sites were identical between studies (Fig. 3). We assessed the average methylation levels of all significant DMCs regardless of directional change (Fig. 4a). Moreover, we averaged methylation levels of the significant DMCs that were consistent in the direction of methylation change (9 increased sites were averaged from the lean and obese study and 29 decreased sites were averaged for the RYGB cohort) (Fig. 4b). Both analyses presented similar average methylation levels between the lean and post-surgery and the obese and pre-surgery (Fig. 4a, b).

## Discussion

Our previous study had identified *SORBS3* as an obesity-associated gene, whose expression may be epigenetically regulated [8]. We set out to further establish the relationship between *SORBS3* methylation and gene expression changes with obesity through a surgical weight-loss intervention. Three months following the RYGB surgery, there were significant reductions in weight and improvement of metabolic measures such as BMI, percent body fat, and fasting plasma insulin levels. However, we did not observe improvements in EGP or *M*-value as determined by the euglycemic-hyperinsulinemic clamp. Our observations are consistent with others [15, 16]. EGP has been shown to significantly decrease immediately after surgery and return to pre-surgery measures at 3 months [15]. The *M*-value has been shown to significantly improve at 12 months with major weight loss [16]. It could be hypothesized that we may have observed improvements in EGP and the *M*-value had we extended the study beyond the 3 months post-surgery.

Environmental factors can influence transcriptional regulation through DNA methylation. Following weight loss induced by surgery, we observed significant decreases in *SORBS3* methylation and increased gene expression. When we applied a conservative Bonferroni correction, we demonstrated two highly significant associations between *SORBS3* DMCs and fasting plasma glucose levels. Moreover, the gene expression data was significantly associated with BMI, percent body fat, and fasting insulin levels. In another study, Barres et al. identified significant positive correlations between *PGC1α* methylation levels and phenotypes, such as BMI and triglycerides 6 months post-surgery [17]. Both the Barres et al. study and our current study have observed that methylation changes are correlated with metabolic phenotypes [17]. This observation would suggest that the methylation changes are not contingent on the alteration of one phenotype, but changes in an individuals’ metabolic status. Collectively, our findings highlight the relationship between decreased *SORBS3* DNA methylation in the presence of weight loss induced by surgery.

The changes observed in *SORBS3* DNA methylation and gene expression complement our previous findings [8]. Interestingly, reduced methylation and increased expression post-surgery were relatively proportional to levels found in our lean individuals, even though the mean BMI for these groups were not comparable. This link between DNA methylation and gene expression changes was further established by in vitro measures. The luciferase assay has been used in this study and in others [17, 18] as a reliable means of providing evidence for the regulatory role of promoter methylation on gene expression. Typically, with the use of multiple methyltransferases, a stepwise decrease in luciferase expression is observed with an increasing number of methylated sites [18]. We observed decreased gene expression with the methylation set by SssI, but not with HhaI, suggesting the number and positioning of the sites in that promoter to be important. Coincidentally, the methylation changes identified in the current study were only represented within the SssI methylated sites and not the HhaI. However, the exact mechanism in which our DNA methylation sites regulate the transcription of *SORBS3* has not been elucidated. We have identified potential transcription factor binding motifs that may be affected by the presence of methylation, but require further investigation. Specifically, the transcription factors that may be influenced in this assay that overlap with our sites of interest are GC-binding factor (GCF), specificity protein 1 (Sp1), and activating enhancer-binding protein 2-alpha (AP-2 Alpha).

The gene *SORSB3* codes for the cytoskeletal adapter protein vinexin [19]. Previously, we have shown that cytoskeletal proteins are reduced in insulin resistance [20]. We have proposed a model in which the reduction in cytoskeletal elements can disrupt the sensing of contractile activity, leading to altered mechanosignaling for gene expression changes in mitochondrial biogenesis [10]. This can potentially lead to a reduction and abnormal function of mitochondria and ultimately result in cellular abnormalities (lipid accumulation, reduced fat oxidation, and insulin signaling) related to insulin resistance. It is tempting to speculate that a reduction in vinexin abundance may play a role in the altered cytoskeletal organization for mechanosignal transduction proposed with insulin resistance. Furthermore, our current findings with weight loss induced by surgery could ameliorate these changes associated with obesity and insulin resistance.

## Conclusions

Collectively, the post-surgery findings present an exciting new addition to understanding the DNA methylation changes associated with *SORBS3* expression. We have previously detected differences associated with *SORBS3* in individuals with obesity and insulin resistance, and the present study provided further evidence of alterations in *SORBS3* in response to weight loss by surgical intervention. However, we acknowledge the limitation of our sample size in the present study. Future studies will need to confirm our findings in a larger cohort. Moreover, we observed in vitro the suppression of *SORBS3* promoter DNA methylation on transcriptional activity. The specific placement of these sites can play an important role on the binding ability of transcription factors. We identified potential transcriptional regulators overlapping our methylation sites; however, follow-up studies will be necessary to refine the specific interaction.

## Abbreviations

BMI: Body mass index
DMC: Differentially methylated cytosine
EGP: Endogenous glucose production
FPG: Fasting plasma glucose
FSI: Fasting serum insulin
HbA1c: Hemoglobin A1c
HDL: High-density lipoprotein
HOMA-IR: Homeostatic model assessment for insulin resistance
LDL: Low-density lipoprotein
*M*-value: Insulin-stimulated glucose disposal
RRBS: Reduced representation bisulfite sequencing
RYGB: Roux-en-Y gastric bypass
*SORBS3*: Sorbin and SH3 domain containing 3
UTR: Untranslated region
